# Descriptive Analysis of Carrier and Affected Hereditary Fructose Intolerance in Women during Pregnancy

**DOI:** 10.3390/healthcare12050573

**Published:** 2024-02-29

**Authors:** Estefanía Zuriaga, Sonia Santander, Laura Lomba, Elsa Izquierdo-García, María José Luesma

**Affiliations:** 1 Facultad de Ciencias de la Salud, Universidad San Jorge, Campus Universitario, Autov A23 km 299, 50830 Villanueva de Gállego Zaragoza, Spain; ezuriaga@usj.es (E.Z.); llomba@usj.es (L.L.); 2 Faculty of Health and Sports Sciences, University of Zaragoza, 22002 Huesca, Spain; 3 Department of Pharmacy, Hospital Universitario Infanta Leonor, 28031 Madrid, Spain; elsaizquierdogarcia@gmail.com; 4 Department of Human Anatomy and Histology, Faculty of Medicine, University of Zaragoza, 50009 Zaragoza, Spain; mjluesma@unizar.es

**Keywords:** pregnancy, hereditary fructose intolerance, physical and emotional state, nutritional habits, development of pathology, baby information

## Abstract

(1) Background: Hereditary fructose intolerance (HFI) is a rare autosomal recessive metabolic disorder resulting from aldolase B deficiency, requiring a fructose, sorbitol and sucrose (FSS)-free diet. Limited information exists on the relationship between pregnancy outcomes and HFI. This study aims to analyze pregnancy-related factors in a cohort of thirty Spanish women, with twenty-three being carriers and seven being HFI-affected (45 pregnancies). (2) Methods: A descriptive, cross-sectional and retrospective study utilized an anonymous questionnaire. (3) Results: Findings encompassed physical and emotional states, nutritional habits, pathology development and baby information. Notable results include improved physical and emotional states compared to the general population, with conventional analyses mostly within normal ranges. Persistent issues after pregnancy included hepatic steatosis, liver adenomas and hemangiomas. Carrier mothers’ babies exhibited higher weight than those of patient mothers, while the weights of carrier children born with HFI were similar to disease-affected children. (4) Conclusions: Pregnant women with HFI did not significantly differ in physical and emotional states, except for nausea, vomiting, and cravings. Post-pregnancy, HFI patients and carriers exhibited persistent hepatic issues. Significantly, babies born to HFI-affected mothers had lower weights. This study sheds light on pregnancy outcomes in HFI, emphasizing potential complications and the need for ongoing monitoring and care.

## 1. Introduction

**Figure 1:**
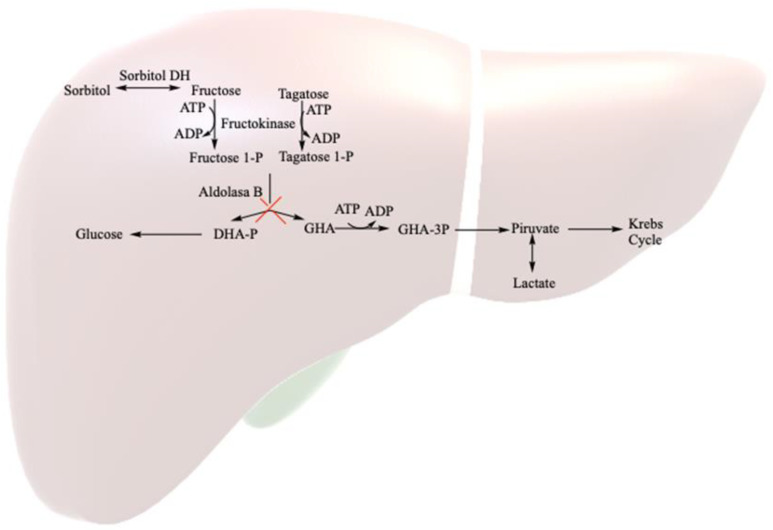
Fructose metabolism in the liver. DHA: dihydroxyacetone; GAH: glyceraldehyde; P: phosphate; DH: dehydrogenase.

Hereditary fructose intolerance (HFI; OMIM 229600) is a rare autosomal recessive inborn error of metabolism, with a prevalence of around 1/20,000, resulting from a deficiency of the enzyme fructose-1,6-bisphosphate aldolase (aldolase B = ALDOB) [1]. This enzyme, crucial in fructose metabolism, is unable to break down fructose 1-phosphate into glycolysis metabolites (mainly in the liver, but also in renal tubules and the small intestine) (Figure 1). Symptoms in homozygous HFI patients vary based on fructose consumption. The consumption of large amounts (4–6 g/kg body weight (bw)/day) leads to rapid symptomatology, including nausea, vomiting, sweating, lethargy, shock, dehydration, hepatic and renal dysfunction and hypoglycemia, and severe cases may progress to coma or death. The continued consumption of small fructose amounts (up to 250 mg/kg bw/day) may result in feeding difficulties, vomiting, hepatomegaly, fatty liver, edema, ascites and failure to thrive [2,3,4].

The sole treatment for HFI patients is a restricted diet, limiting total fructose intake to less than 40 mg/kg body weight (bw) per day. Naturally occurring fructose in fruits and vegetables, as well as sweeteners like saccharose (fructose and glucose disaccharide) and honey, poses challenges. Sorbitol is converted to fructose by the human body through sorbitol dehydrogenase, and it is found in other polyols like maltitol, lactitol or isomaltitol. Additionally, sweeteners like tagatose, metabolized by aldolase B, are not recommended for HFI patients [5].

Heterozygous carriers of HFI are relatively common in the general population, with a predicted frequency ranging from 1:55 to 1:122. These carriers typically do not adhere to a restricted fructose diet, as it is presumed that a 50% level of ALDOB activity is adequate for proper functioning [6,7]. Studies indicate that carriers experience no significant alterations in postprandial fructose metabolism when on a low-fructose diet (<10 g/day), with only an observed increase in plasma uric acid concentration [8]. However, metabolic changes become more pronounced with a high fructose diet (50–100 g/day), leading to increased postprandial uric acid, insulin concentration and hepatic insulin resistance index [9].

Pregnancy introduces various physical, emotional and metabolic disturbances, prompting changes in habits such as diet, alcohol, tobacco or medication consumption and physical activity in the general population. These aspects are not well-understood in HFI patients and carriers. Consequently, this study aims to analyze items related to pregnancy and newborns in a cohort of Spanish HFI patients and asymptomatic carriers of HFI.

This study on HFI explores pregnancy outcomes in women with aldolase B gene mutations, shedding light on the rare disorder’s impact on reproductive health. The focus on dietary management, genetic basis and complications during and after pregnancy provides valuable insights into the challenges faced by these women. Additionally, this study underscores the significance of understanding how HFI influences offspring. Overall, this research informs healthcare professionals about the specific considerations and complications associated with pregnancy in HFI, guiding tailored care for this rare metabolic disorder.

## 2. Materials and Methods

### 2.1. Study Design

This study employed a descriptive, cross-sectional and retrospective design to elucidate the perceptions and habits during pregnancy among women affected by HFI and carriers of the condition and to gather information regarding their infants, while also exploring correlations between these phenomena and the individual’s status as a carrier of or being affected by the disease.

### 2.2. Participants

The study population comprised women who were either carriers of or affected by hereditary fructose intolerance (HFI) and had experienced at least one pregnancy. The sample was recruited through non-probabilistic snowball sampling, facilitated by the Spanish Association of People Affected by Hereditary Fructose Intolerance. Women meeting the inclusion criteria and expressing voluntary participation were included in the study. Inclusion criteria encompassed Spanish nationality, a history of full-term pregnancy and a proficient understanding of the questionnaire’s language. Additionally, HFI-affected women required a confirmed diagnosis through methods such as liver biopsy–enzyme assay, fructose challenge test or molecular genetic analysis. HFI carriers could provide confirmation through molecular genetic analysis (heterozygous ALDOB mutation/deletion) or by having a son or daughter with a confirmed diagnosis. 

### 2.3. Data Collection

**Figure 2:**
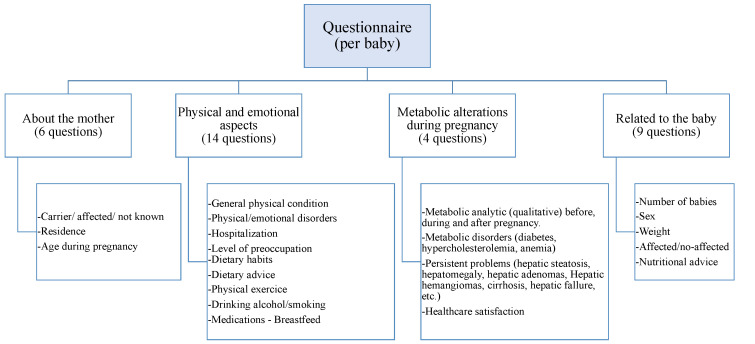
Questionnaire sections.

For this study, an anonymous questionnaire was developed and employed as a data collection tool through Google Forms^®^ (online survey services). The questionnaire design drew inspiration from the “Perception and habits of Spanish women during pregnancy” study [10], conducted in April 2018 on a sample of 2436 Spanish mothers with children under 4 years old. Questions were adapted to our population while preserving the structure and semantics for validity, and additional inquiries related to HFI were included. The final survey, comprising 33 questions, covered various sections, including sociodemographic details, physical and emotional aspects of the mother during pregnancy, clinical information (including medications and nutritional habits) and issues related to the newborn (Figure 2).

Closed questions covered sociodemographic details, the number of hospital admissions or questions about the baby. Questions related to illness, nutritional habits, professional advice, satisfaction with the health system, exercise, smoking, alcohol, drug habits and breastfeeding during pregnancy followed a dichotomous format (yes/no or good/bad). Biochemical analysis questions allowed three response options (low, normal, or high), and Likert-type format questions with five possible answers assessed the general state of health and level of concern, accepting only one response per question. Open questions covered nutritional habits, newborn’s weight and size, and general aspects. Women with multiple pregnancies completed one survey for each pregnancy.

The study information and questionnaire link were electronically disseminated through the Spanish HFI Association via email (asociacionihf@gmail.com) or mobile phone. The questionnaire remained open for 8 weeks (September–October 2022), with a reminder sent midway to boost participation.

### 2.4. Data Analysis

Descriptive results derived from the questionnaire were expressed as measures of central tendency (mean and standard deviation) for quantitative variables and as relative and/or absolute frequencies for qualitative variables. To explore correlations and differences between variables in the HFI-affected and carrier groups, various statistical tests were employed. The chi-square test and Fisher’s exact test were applied for comparing qualitative variables, while Student’s *t*-test or non-parametric tests (in case of non-normality) were utilized for quantitative variables. A significance level of *p* < 0.05 was considered statistically significant. The SPSS v27.0 program facilitated the statistical analysis. The study is limited by the low number of affected and carrier populations. Since this is a rare disorder, precision was estimated as a percentage and a 95% confidence interval was estimated using EPIDAT 3.1 software to show the power of the sample. 

### 2.5. Ethical Aspects 

The study received approval from the Aragón Ethics Committee for Investigation (CEICA) (C.P.—C.I. PI22/368. Acta Nº 14/2022). CEICA determined that:

- The project aligns with the requirements of Spanish Law 14/2007 on biomedical research, and its implementation is relevant.
- The protocol meets the necessary criteria for suitability concerning the study objectives, justifying foreseeable risks and discomforts for the subjects.
- The use of data and documents for obtaining consent is deemed appropriate.
- The economic compensation provided does not compromise adherence to ethical principles.
- The researchers possess the required capacity and resources to conduct the study.

Participants were required to read and accept an informed consent and privacy policy from Zaragoza University and Google^®^ before participating in the survey.

## 3. Results

A total of 30 women, including 23 carriers and 7 affected by HFI, provided survey responses for each of their pregnancies, with 13 of them reporting information on multiple pregnancies (12 with two children and 1 with three children), resulting in a total of 45 responses. These women were born between 1954 and 1991 (median value 1974), representing a range of decades that have witnessed the evolving knowledge of the HFI disease. The mean age of women at delivery was 32.1 (SD = 3.88), with a minimum age of 23 years old and a maximum age of 39.

The results are presented for each pregnancy according to the different areas of study explored with the questionnaire. 

### 3.1. Physical and Emotional State of Pregnancy

The majority of respondents (91.1%) reported being in good physical condition during pregnancy, while 8.9% of pregnancies, all from carrier women, indicated poor physical condition. With a confidence of 95%, these results showed 10.54% precision in the estimation of poor physical condition and a 95% confidence interval of 2.5–21.2. Similar percentages were observed for the emotional state. However, pregnancies with poor physical condition were distinct from those with poor emotional states.

Table 1 outlines the survey results related to the physical and emotional states of pregnancy. Digestive disorders, nausea and vomiting were the prevalent physical symptoms in HFI carrier women, with a strikingly low frequency in HFI-affected women. Conversely, symptoms such as tired and swollen legs and fluid retention were more frequent in women affected by HFI. A statistically significant difference was found between HFI carriers and affected groups concerning “nausea and vomiting” symptoms (*p* < 0.001). Although not statistically significant, a slight trend was observed for digestive disorders (*p* = 0.095) and vaginal infections (*p* = 0.068).

Additionally, tiredness, fatigue or drowsiness emerged as the most frequent emotional symptoms in pregnancies of HFI carrier women, contrasting with cravings observed in HFI-affected women. A statistically significant difference was found between HFI carriers and affected groups regarding “cravings” symptoms (*p* = 0.004). Although not statistically significant, a slight trend was noted for decreased self-esteem due to physical appearance (*p* = 0.056).

The mean and standard deviation of Likert-type responses regarding the emotional state during pregnancy are presented in Table 2. The data are categorized based on information from HFI carriers and HFI-affected women. Notably, the health of the baby, childbirth and early care were the most concerning aspects for both groups of women.

### 3.2. Nutritional Habits during Pregnancy

Changes in general dietary habits during pregnancy did not exhibit significant differences between carriers and those affected by HFI. HFI patients follow an FSS-free diet to manage their condition; however, carriers of HFI do not need to adhere to FSS-restricted diets. According to this study, 66.70% of pregnant women (47.60% carriers and 19.0% affected) maintained their regular eating habits without changing their diet. Furthermore, 68.20% (56.80% carriers and 11.40% affected) opted to avoid problematic foods to minimize risks to both them and the baby. These individuals adhered to recommended water intake and consumed natural, nutrient-rich foods beneficial for fetal development, with percentages of 40.00% (27.50% carriers and 12.50% affected) and 40.50% (31.00% carriers and 9.50% affected), respectively. Additionally, 28.60% (23.80% carriers and 4.80% affected) adopted a pattern of consuming smaller, more frequent meals. Only 17.10% (12.20% carriers and 4.90% affected) specifically consumed foods intended for pregnant women, while a mere 4.90% (2.45% carriers and 2.45% affected) reported unrestricted eating without monitoring quantity and quality.

Analyzing modifications in specific nutritional habits, 47.70% (40.90% carriers and 6.80% affected) indicated stopping the consumption of certain foods, and 14.30% (7.15% carriers and 7.15% affected) started consuming foods during pregnancy that were not part of their regular diet. Examining changes in consumption across various food groups, the percentages of mothers modifying their intake of vegetables, meat, fish or dairy products were 18.20% (11.40% carriers and 6.80% affected), 18.60% (9.30% carriers and 9.30% affected), 19.10% (14.30% carriers and 4.80% affected) and 15.90% (13.60% carriers and 2.30% affected), respectively. Changes in the consumption of eggs and egg products were modest, at around 4.70% (only carriers). Foods such as legumes, nuts and cereals underwent minimal modifications, with values hovering around 2.3% (only carriers). No statistically significant differences were identified between carriers and affected individuals in any of these aspects.

### 3.3. Other Noteworthy Maternal Data

#### 3.3.1. Development of Own Pathologies and Analyses

The parameters examined in conventional analyses exhibited normal ranges before, during and after pregnancy, except those associated with fat metabolism. In this context, two respondents (one carrier and one affected) displayed elevated cholesterol levels before pregnancy, six displayed elevated cholesterol during pregnancy (five carriers and one affected) and four maintained elevated levels after pregnancy (three carriers and one affected). Specifically, one carrier had increased LDL cholesterol levels before pregnancy, while four carriers experienced an increase during and after pregnancy. Another carrier reported an elevated HDL cholesterol level before pregnancy, with two carriers showing values below the normal range after giving birth. Triglycerides slightly increased during pregnancy for one carrier, and she maintained levels above the normal range post-pregnancy.

Three pregnant women (two carriers and one affected) exhibited higher glucose levels, indicating the development of gestational diabetes, and three (two carriers and one affected) experienced a decline in iron levels. 

Regarding persistent health issues, two HFI patients reported liver-related problems—one (affected) had steatosis before her first pregnancy, and the second (also affected) had steatosis and hepatomegaly before her only pregnancy. The second affected patient developed adenomas and hemangiomas after pregnancy. Among carriers, only one reported liver problems (steatosis, hepatomegaly, adenomas, hemangiomas, cirrhosis and liver failure) after her second pregnancy, along with other enduring digestive, neurological and renal issues. Two other carriers reported steatosis many years after their pregnancies, making it challenging to attribute it directly to the pregnancy process. In terms of non-liver-related problems, one carrier reported the onset of atrial fibrillation 18 months after her pregnancy.

In many cases, these findings were discovered some time after pregnancy, so those who answered ‘after’ were unaware whether these alterations occurred during or sometime after, as in many cases, medical tests such as liver ultrasound or hepatic magnetic resonance imaging were not conducted in the months following childbirth.

#### 3.3.2. Medication Consumption

Upon analyzing results related to drug consumption, no significant differences were observed between carriers and those affected by HFI. Given the common use of these drugs during pregnancy, the most frequently utilized medications included folic acid, paracetamol, iron or multivitamins. Notably, 33.3% (22.20% carriers and 11.1% affected) did not require medication during pregnancy, while 44.0% (35.6% carriers and 8.90% affected), representing 20 respondents, followed clear medication guidelines. Additionally, 15.6% (11.15% carriers and 4.45% affected) had concerns and sought advice from health professionals. Lastly, 2.2% (only carriers) had doubts but continued medication, while 4.4% (only carriers) discontinued their medication. Although the number is not substantial, two respondents chose not to take medication due to lingering doubts. To address these uncertainties, respondents sought guidance from various professionals, with gynecologists and midwives being the primary sources for resolving queries.

#### 3.3.3. Health Recommendations and Lifestyles

One aspect analyzed in this study is the mother’s lifestyle during pregnancy, particularly focusing on physical activity. The data collected from 34 carrier responses and 11 affected responses revealed that 31.1% of women (22.20% carriers and 8.90% affected) refrained from any exercise, while 46.7% (35.60% carriers and 11.10% affected) engaged in moderate exercise, and 2.2% (only affected) pursued intense physical activity. Additionally, 20% (17.80% carriers and 2.20% affected) participated in specific pregnancy-related activities.

The study also delved into the consumption of tobacco and alcohol. From the 45 responses (34 carrier responses and 11 affected responses), it is noteworthy that 80% (66.70% carriers and 13.30% affected) never used tobacco, and 62.20% (42.20% carriers and 20.00% affected) abstained from alcohol. During pregnancy, 8.90% (4.45% carriers and 4.45% affected) and 37.80% (33.30% carriers and 4.40% affected) discontinued tobacco and alcohol consumption, respectively. Furthermore, 11.10% (4.40% carrier and 6.70% affected) used tobacco during pregnancy, and none of the respondents initiated the use of these substances during pregnancy.

Lastly, the results related to breastfeeding are presented. Notably, there are no significant differences between pregnant women with the disease and those affected. Of the respondents, 15.6% (8.90% carrier and 6.70% affected) did not breastfeed, 17.8% (15.60% carrier and 2.20% affected) breastfed during the first three months, 22.2% (15.60% carrier and 6.70% affected) between 3–6 months, 26.7% (17.80% carrier and 8.90% affected) breastfed for 6–12 months and 17.8% (only carrier) continued breastfeeding for more than 12 months.

#### 3.3.4. Baby Information

Various details pertaining to the babies were obtained from the analysis of 45 pregnancies. Out of these, 91.5% resulted in the birth of a single baby, while 8.5% involved multiple births, yielding a total of 47 babies, with 57.4% being female and 42.6% male. Information on the frequency of single and multiple births, differentiated according to carriers and women affected by IHF, is given in Table 3.

As observed in Table 3, term births were predominant compared to pre-term births and births occurring after 42 weeks. The average weight of a baby was 3322 g (SD = 652), ranging from a minimum of 1800 g to a maximum of 5010 g. Specifically, the average weight of carrier children was 3413 g (SD = 664), and for affected children, it was 3058 g (SD = 560). A significant difference in baby weight was observed between carriers and patients, with babies of carrier mothers having a higher weight. The weight of carrier children born with the disease was 3440 g, while those born without the disease weighed 3490 g. Regarding height, a total of 34 responses were obtained; the median height was 50.5 cm (SD = 2.95), with 43.0 cm being the minimum and 56.0 cm the maximum height.

Only one HFI-affected individual had a child with the disease, compared to 23 sick children born to carrier mothers, as can be observed in Table 3. Additionally, results related to responses regarding HFI disease in each baby are presented. For HFI carrier babies, it is noteworthy that 35% of them were uncertain about their baby’s carrier status. When analyzing problems related to babies during the disease, it is observed that 23 babies (48.9%) were affected by HFI, 8 babies (17%) were carriers of the disease and 16 do not know or do not answer (34.1%). Furthermore, 26.5% of babies experienced some health issues, and 13 babies (32.50%) required nutritional counseling for children.

Moreover, reported health problems in babies included liver issues, high cholesterol, constipation, gas, hypertransaminasemia, hepatomegaly, steatosis, gastrointestinal disorders, hypoglycemia, celiac disease, nephrotic syndrome, growth retardation and respiratory problems, among others.

## 4. Discussion

To the best of our knowledge, the pathogenesis of pregnant HFI patients or carriers has not been explored in depth beyond the recommended follow-up care during pregnancy [11]. One of our objectives was to understand the physical symptoms and potential health complications in this population. In pursuing this, we utilized a survey conducted in 2018 by the pharmaceutical laboratory CINFA [10] as a reference, aiming to comprehend the experiences of Spanish women during pregnancy concerning their main symptoms, concerns, emotional state and the social support received. More information about the parameters or criteria used for benchmarking against the general population can be found in the CINFA Survey (http://cdn-cinfasalud.cinfa.com/wp-content/uploads/2018/10/Dossier-Estudio-CinfaSalud-Embarazo_abril18.pdf, accessed on 2 January 2023).

Noteworthy is the absence of significant differences in physical and emotional status between HFI carriers and those affected. In fact, some aspects even surpass those found in the general population. Regarding the subjective perception of physical well-being during pregnancy, 91.1% of our study’s participants claimed to have felt well, contrasting with 83.3% of pregnant women in the CINFA survey [10]. Physical symptoms, such as digestive disorders, nausea, vomiting, tired and swollen legs and various other issues (back and skin problems, olfactory and taste disturbances, constipation, gingivitis, pain and itching in the breasts, pelvic pain, urine leakage, varicose veins and spider veins, abnormal weight gain, urinary tract infections, migraines and headaches, respiratory distress, vaginal infections, metacarpal tunnel, punctures and early contractions with threat of premature labor and tachycardia), showed comparable frequencies in our population compared to the CINFA survey results. Notably, our participants exhibited a higher incidence of hemorrhoids and vaginal bleeding before childbirth. However, no bibliographical references have been found to correlate these symptoms with the disease.

Digestive disorders (55%), along with nausea and vomiting (46.6%), were the most prevalent physical symptoms among pregnant women in the CINFA survey [10], while among HFI mothers and carriers, they were nausea and vomiting (59.5%), hemorrhoids (55.6%) and digestive disorders (48.8%). A significant difference in the frequency of nausea and vomiting was observed between carriers and affected women. Carriers exhibited a higher frequency of nausea and vomiting compared to the affected patients, warranting further investigation.

Despite being less disabling than other rare diseases, over 50% of the Spanish HFI population reported no problems in any quality-of-life dimension. However, some encountered difficulties in daily activities, experienced pain and reported anxiety [12].

In terms of conventional analysis parameters, most parameters studied were within a normal range before, during and after pregnancy, except those related to fat metabolism. It is essential to highlight that even HFI patients under a fructose-restricted diet may exhibit higher intrahepatic fat content [13]. 

Two of the respondents (one carrier and one affected) presented elevated cholesterol levels prior to pregnancy. This figure increased to six during and after pregnancy (five carriers and one affected), with four individuals sustaining elevated blood cholesterol levels (three carriers and one affected). Specifically, one carrier exhibited elevated LDL cholesterol levels before pregnancy. During pregnancy, LDL cholesterol increased in four carriers, and these levels persisted after pregnancy. Regarding HDL cholesterol, one carrier reported an increase before pregnancy, but it did not seem to be further affected during the course of pregnancy. Remarkably, two carriers even reported values below the normal range after giving birth. In pregnancy, hormonal changes are associated with major changes in the lipid profile and a moderate variation in cholesterol and triglyceride levels is considered physiological [14,15]. In our study, it is not possible to determine the extent of variation in these parameters above the normal reference values in the general population.

The magnetic resonance imaging spectroscopy of the liver revealed that intrahepatic triglyceride (IHTG) content was higher in patients with HFI compared to healthy age, sex and BMI-matched individuals [16]. This is consistent with our observations that triglycerides appeared to have increased slightly during pregnancy in one woman who maintained levels above the range after pregnancy. The increase in hepatic triglycerides is partly explained because de novo lipogenesis is greater with aldolase deficiency. The accumulation of fructose 1 phosphate induces GKRP–GCK dissociation, and glucokinase stimulates hepatic glucose uptake, which is then transformed into glycogen and fat through de novo lipogenesis [14,15].

Hepatic fat accumulation persists in HFI patients, despite a fructose-restricted diet [17]. A recent cross-sectional observational study, encompassing 16 genetically diagnosed HFI patients, reported a high prevalence of fatty liver, as assessed by ultrasound or hepatic magnetic resonance imaging [2]. This could be explained by the endogenous production of fructose through the sorbitol–aldose reductase pathway, activated under specific physiological and non-physiological circumstances. For instance, this pathway is activated after a meal rich in complex carbohydrates, following the administration of nephrotoxic medications, during the sepsis processes or following cardiovascular surgery [18]. Liver steatosis develops due to imbalanced lipid metabolism, characterized by increased rates of de novo lipogenesis and hepatic fatty acid uptake, coupled with reduced fatty acid oxidation and/or disposal to the circulation. Emerging data suggest that dietary fructose may directly influence the expression of genes involved in lipid metabolism, promoting hepatic fat accumulation or impeding fat removal [19]. Non-alcoholic liver steatosis affecting HFI can progress to cirrhosis and later to hepatocellular carcinoma. Despite this, hepatic steatosis is a benign, potentially reversible condition and does not necessarily lead to irreversible liver damage [2,20]. This is consistent with our study, since, in relation to persistent problems associated with HFI patients, we can highlight the presence of hepatic steatosis in two affected individuals before pregnancy (one also with hepatomegaly) and one carrier after pregnancy, and two more carrier women who suffer from steatosis but cannot be temporarily linked to pregnancy. All four exhibited transaminase levels above the normal range.

Two respondents (one carrier and one affected) developed liver adenomas and hemangiomas after pregnancy, a phenomenon reported in the literature associating hepatic adenomas with hepatic steatosis in HFI [21,22]. Liver cirrhosis and failure and severe gastrointestinal, neurological and kidney problems were also observed in the carrier, consistent with literature on HFI patients [23,24] but not carriers. 

None of the women exhibited consistent signs of liver fibrosis. However, one carrier indicated the presence of cirrhosis after pregnancy, aligning with findings from a study that comprehensively examined 15 adult patients with HFI. This study involved individuals on a lifelong fructose-restricted diet and included a control group of 15 participants. Notably, the analysis of liver stiffness, utilized as a non-invasive marker for liver fibrosis, revealed no significant differences between the two groups [16].

Elevations in glucose levels were observed in three pregnant women, leading to the development of gestational diabetes. Additionally, a reduction in iron levels was noted in three other pregnant women, both phenomena being common occurrences during pregnancy. Metabolic profiling indicated that patients with HFI exhibited a higher degree of glucose intolerance [16], suggesting a potential association between these metabolic alterations.

Changes in dietary fructose levels profoundly affect gut microbiota, leading to a microbiome with altered metabolic capacity. This may have implications for intestinal fructose absorption, luminal concentrations and the microbiome composition in HFI patients [9,25,26], presenting an avenue for future research.

Vitamin deficiencies are a concern due to the lifelong fructose-, sucrose- and sorbitol-restricted diet in HFI patients. Supplementation with vitamin C and folic acid has been explored [27], and it is crucial to maintain correct vitamin status in pregnant women with HFI. Circulating levels of vitamin C are inversely associated with metabolic syndrome [28]. Folate deficiency typically arises from malabsorption disorders, hemolytic anemia, increased requirements during pregnancy or a diet lacking in this compound [28]. Cano et al. [29] demonstrated in their study that the majority of HFI participants exhibited dietary intake levels below the recommended population levels for vitamin C (96.7%) and folate (90%). This study, for the first time, provides evidence supporting vitamin C supplementation in individuals with HFI. It would be worthwhile to explore optimal doses of vitamin C and folic acid supplementation for HFI patients or carriers, both in normal conditions and during pregnancy.

There was a significant disparity in the birth weights of infants born to carriers and those born to HFI patients, with babies of carrier mothers, some of whom were affected by HFI, exhibiting higher weights. Furthermore, the results indicate a noteworthy similarity in the birth weights of carriers’ children, irrespective of whether they were born with the disease or not. This observation supports the notion that infants born to HFI carriers are generally born healthy.

Although fructose transporters (GLUT-2 or GLUT-5) have not been identified in the placenta, there is a possibility that a minimal amount of fructose may pass through non-transporter-mediated diffusion mechanisms [30]. However, this is believed to occur to a much lesser extent than the passage of glucose [31]. In fetal tissues, aldolase A predominates, but during embryonic development, its expression is suppressed in the liver, kidney and intestine, concomitant with the upregulation of aldolase B. The extent to which fructose crosses the placenta and the expression of aldolase B in the fetus remain unknown. However, it is noteworthy that HFI patients manifest their first symptoms shortly after the introduction of fructose or sucrose into their diet, rather than at birth [17].

The inadequate restriction of fructose in the diet can lead to growth deficits. Despite a brief period of oral nutrition, instances of growth retardation have been documented in these patients [32,33]. The observation that infants born to affected women exhibit lower birth weights, even when not affected by the disease, merits further investigation to elucidate any potential correlation between the maternal condition and fetal development.

This study has several limitations, primarily related to self-administered surveys where users respond to clinical and health questions based on their knowledge. Additionally, retrospective cases may be subject to recall bias, as some episodes referred to events that occurred many years ago. The inability to collect all analytical data from participants due to missing information further contributes to the study’s limitations. A specific limitation of research in rare diseases is always the small sample size. In our case, despite the small sample size, the percentage of women who reported poor pregnancy outcomes could be estimated with acceptable precision. Also, due to the small sample size, no adjustments were made for confounding variables, which could further reduce the sample size and make the interpretation of the results difficult. Although this task is left to future studies with larger sample sizes, the authors believe that our results provide valid information for the appropriate management of women with fructosemia during pregnancy.

## 5. Conclusions

This study involved a survey focusing on the pregnancy experiences of women who are carriers of and affected by HFI. A descriptive study design was employed to analyze various aspects related to pregnancy and babies in this specific population.

In the case of pregnant women, our observations suggest that the physical and emotional statuses between carriers and those affected by HFI do not exhibit significant differences, except in symptoms related to nausea, vomiting and cravings. In terms of analytical parameters, it is noteworthy that most of the parameters studied, as gathered through the survey, fall within a random range of normality before, during and after pregnancy. However, exceptions include those related to fat metabolism and increases in glucose levels. Furthermore, persistent problems associated with HFI patients and carriers include hepatic steatosis, liver adenomas, and liver hemangiomas after pregnancy.

Concerning babies, our findings reveal significant differences in the weight of babies born to carriers compared to babies born to HFI patients, with carrier mothers having babies with higher weights.

Despite the valuable insights gained from this study, it is crucial to acknowledge the need for expanding the analyzed population. Given the rarity of the disease, the available sample size is limited. However, the study provides relevant information that warrants further exploration in subsequent research endeavors.

This study on HFI provides crucial insights into improving care for carriers and affected women during pregnancy. Despite improved physical and emotional states compared to the general population, persistent post-pregnancy hepatic issues, notably hepatic steatosis, liver adenomas, and hemangiomas, were observed in HFI patients and carriers. Babies born to HFI-affected mothers had lower weights, emphasizing the need for ongoing monitoring. Future studies should explore optimal vitamin supplementation for HFI patients, address birth weight disparities and delve into the correlation between maternal condition and fetal development. This research underscores the importance of tailored care, continuous monitoring and potential interventions to enhance the standard of care for pregnant women with HFI. 

## Figures and Tables

**Table 1:** Absolute and relative frequencies of “yes” responses in the results of the physical and emotional states of pregnancy, divided into HFI carriers and affected women.

Symptoms; (N = HFI Carriers; HFI Affected)	Absolute (Relative) Frequencies; n (%)	
	HFI Carrier	HFI Affected
Digestive disorders (N = 30; 11)	17 (41.5%)	3 (7.3%)
Nausea and vomiting (N = 32; 10)	25 (59.5%)	0 (0.0%)
Tired and swollen legs or retained fluids (N = 26; 11)	8 (21.6%)	5 (13.5%)
Back problems (N = 28; 10)	8 (21.1%)	1 (2.6%)
Changes in the skin (N = 29; 10)	12 (30.8%)	2 (5.1%)
Olfactory and taste alterations (N = 26; 10)	4 (11.1%)	2 (5.6%)
Constipation (N = 28; 9)	11 (29.7%)	2 (5.4%)
Hemorrhoids (N = 26; 10)	12 (33.3%)	4 (11.1%)
Gingivitis problems or inflammation/bleeding gums (N = 25; 10)	1 (2.9%)	2 (5.7%)
Pain and itching in the breasts (N = 26; 10)	3 (8.3%)	1 (2.8%)
Pelvic pain (N = 25; 10)	6 (17.1%)	1 (2.9%)
Urine leakage (N = 26; 10)	4 (11.1%)	1 (2.8%)
Varicose veins and spider veins (N = 27; 10)	8 (21.6%)	1 (2.7%)
Weight gain outside of normal parameters (N = 27; 11)	6 (15.8%)	1 (2.6%)
Urinary infections (N = 27; 10)	1 (2.7%)	1 (2.7%)
Migraines and headaches (N = 27; 10)	0 (0.0%)	0 (0.0%)
Difficulty breathing (N = 28; 10)	3 (7.9%)	0 (0.0%)
Vaginal bleeding before the onset of labor (N = 28; 11)	8 (20.5%)	2 (5.1%)
Vaginal infections (N = 27; 10)	0 (0.0%)	2 (5.4%)
Wrist pain (metacarpal tunnel) (N = 25; 10)	0 (0.0%)	0 (0.0%)
Punctures and early contractions with threat of premature labor (N = 27; 10)	2 (5.4%)	1 (2.7%)
Tachycardia, arrhythmias, etc. (N = 27; 10)	0 (0.0%)	0 (0.0%)
Tiredness, fatigue or drowsiness (N = 34; 11)	19 (42.2%)	6 (13.3%)
Emotional ups and downs (N = 33; 11)	10 (22.7%)	3 (6.8%)
Insomnia (N = 29; 11)	6 (15.0%)	2 (5.0%)
Fears and concerns (N = 32; 11)	10 (23.3%)	3 (7.0%)
Continuous feeling of hunger (N = 30; 11)	2 (4.9%)	3 (7.3%)
Decreased sexual appetite (N = 30; 11)	10 (24.4%)	4 (9.8%)
Increased sexual appetite (N = 29; 10)	3 (7.7%)	0 (0.0%)
Cravings (N = 29; 11)	5 (12.5%)	7 (17.5%)
Anxiety (N = 29; 10)	1 (2.6%)	0 (0.0%)
Decreased self-esteem due to physical appearance (N = 29; 11)	1 (2.5%)	3 (7.5%)
Depressed mood (N = 29; 11)	1 (2.5%)	1 (2.5%)

**Table 2:** Mean and standard deviation (SD) from results of the emotional state of pregnancy questions with a Likert-type format divided into HFI carriers and affected women. There were five possible answers from never, 1, to almost always, 5.

Symptoms; (N = HFI Carriers; HFI Affected)	HFI Carrier		HFI Affected	
	Mean	SD	Mean	SD
Baby’s health (N = 34; 11)	3.65	0.950	3.27	1.19
Childbirth (N = 34; 11)	3.18	1.14	3.36	1.03
Baby and first care (N = 32; 11)	3.03	1.20	2.73	1.10
Not being able to breastfeed (N = 32; 10)	2.44	1.24	2.50	1.08
Effect of pregnancy on maternal health (N = 32; 11)	2.16	0.920	2.36	0.924
Baby and family economy (N = 32; 11)	1.71	0.824	2.09	0.944
Postpartum depression (N = 32; 11)	2.16	1.14	2.00	1.34
Effect on relationship (N = 32; 11)	1.94	0.878	2.18	1.33
Baby effect at professional development (N = 31; 11)	1.97	1.05	2.36	1.21
Long-term physical effect (N = 32; 11)	1.94	0.914	2.18	1.33
Social level effect (N = 32; 11)	1.75	0.803	2.36	1.29
Sex life effect (N = 32; 11)	1.75	0.842	2.36	1.21

**Table 3:** Absolute and relative frequencies of the baby and birth information, divided into HFI carriers and affected women.

Baby and Birth Information; (N = Total Number of Births or Babies with the above Characteristic)	Absolute (Relative) Frequencies; n (%)	
	HFI Carrier	HFI Affected
Birth of a single baby (N = 43)	33 (70.20%)	10 (21.30%)
Multiple birth (N = 4)	2 (4.30%)	2 (4.30%)
Term births (N = 38)	30 (63.80%)	8 (17.00%)
Pre-term births (N = 5)	3 (6.40%)	2 (4.30%)
Post-term birth (N = 4)	2 (4.30%)	2 (4.30%)
HFI-affected baby (N = 23)	22 (48.9%)	1 (2.20%)

## Data Availability

Data are contained within the article.
